# Supplementary material for: Distance-decay reveals contrasting effects of land-use types on arthropod community homogenisation
Source: Nat Commun. 2026 Jan 15;17:763. doi: 10.1038/s41467-025-67612-9 (PMC12820165; doi:10.1038/s41467-025-67612-9)
Supplement: Supplementary file 1 — Supplementary Information [file 41467_2025_67612_MOESM1_ESM.pdf]

Supplementary Information

Note 1.

α-diversity: Includes diversity metrics describing species similarities or differences within an assemblage or sample.

β-diversity: Includes diversity metrics describing species similarities or dissimilarities between particular assemblages or samples. β-diversity is calculated by $\alpha \div \gamma$ -diversity.

γ-diversity: Includes total species and refers to species across many assemblages or samples. γ-diversity pools together species from a broad region or landscape.

Distance-decay: A type of β-diversity describing community similarity change with distance. Distance-decay compares species similarity of communities over a physical distance gradient. Distance-decay curve is calculated using community species similarity on the y-axes and community physical distance on the x-axes. Species communities physically located close to each other should be more similar than communities which are far away from each other. When species communities are highly similar to each other despite their physical distance, the distance-decay is weak (x-y curve flat); and when the communities are not similar even though they are physically close together, the distance-decay is strong (x-y curve steep). Biotic homogenisation of species is assumed when distance-decay is weak and species composition in a community is similar, independent of the physical distance between. In our study we used taxonomic diversity to calculate distance-decay.

Community homogenisation and heterogenisation: During a specific process, such as land-use intensification, species composition in a given two (or more) communities can become less or more similar to each other taxonomically or functionally. In our study, we only use taxonomical differences. This way, when communities are sharing more of the same species, they are homogenised, therefore communities are going through species homogenisation. On the other hand, when communities are sharing less of the same species, we consider this process species heterogenisation. Based on the idea of distance-decay, when distance-decay is weak, the communities are homogenised, and when distance-decay is strong, communities are heterogenised.

Supplementary Table 1. The output of SIMBA analysis. The analysis is testing slope differences by calculating the statistical inference of the difference in slope between two regression lines and adjusting for the multiple comparisons with Bonferroni-Hochberg method. Comparisons are made between local land-use types. Communities with different Hill numbers are separated (q0, q1, q2). Bold letters indicate a significant effect ($p < 0.05$). Slopes ± SE of each local land-use distance-decay were extracted from the linear regression models.

Hill-nr.	Comparison *land-use*	Difference in slope	Adjusted p (BH)
	Arable land-Forest	2.4E-05	0.996
	Arable land – Grassland	9.8E-05	0.006
	Arable land-Settlement	7.6E-06	0.999
	Grassland – Forest	7.4E-05	0.006
	Settlement-Forest	3.1E-05	0.648
q0	Settlement-Grassland	1.1E-04	0.006
		Slope	SE
	Arable land	-2.5E-04	2.2E-05
	Forest	-2.2E-04	1.6E-05
	Grassland	-1.5E-04	2.0E-05
	Settlement	-2.5E-04	2.5E-05
	Arable land-Forest	5.0E-05	0.186
	Arable land – Grassland	1.0E-04	0.006
	Arable land-Settlement	2.3E-06	0.999
	Grassland – Forest	5.2E-05	0.132
	Settlement-Forest	5.2E-05	0.162
q1	Settlement-Grassland	1.0E-04	0.006
		Slope	SE
	Arable land	-2.9E-04	2.3E-05
	Forest	-2.4E-04	1.7E-05
	Grassland	-1.9E-04	2.1E-05
	Settlement	-2.9E-04	2.6E-05
	Arable land-Forest	6.8E-05	0.036
	Arable land – Grassland	1.2E-04	0.006
	Arable land-Settlement	1.5E-05	0.999
	Grassland – Forest	5.2E-05	0.114
	Settlement-Forest	5.3E-05	0.138
q2	Settlement-Grassland	1.1E-04	0.006
		Slope	SE
	Arable land	-3.1E-04	2.4E-05
	Forest	-2.4E-04	1.7E-05
	Grassland	-1.9E-04	2.1E-05
	Settlement	-3.0E-04	2.7E-05

Supplementary Table 2. Table showing the output of the Tukey's post-hoc test, testing the community weighted means of body size and mobility in each local land-use type. Estimate, degrees of freedom, t-ratio and p-value (or Tukey's Honest Significant Difference) is shown for each comparison. Bold values indicate significant differences ($p < 0.05$).

Trait	Comparison	Est.	DF	t-ratio	p-value
Body size	Forest - Arable land	0.136	175	1.57	0.340
	Grassland - Arable land	-0.090	175	-0.99	0.750
	Settlement - Arable land	-0.218	175	-2.26	0.111
	Grassland - Forest	-0.225	175	-2.63	0.045
	Settlement - Forest	-0.353	175	-3.84	0.001
	Settlement - Grassland	-0.128	175	-1.33	0.541
Mobility	Forest - Arable land	-0.011	175	-2.53	0.014
	Grassland - Arable land	-0.013	175	-2.72	0.034
	Settlement - Arable land	-0.012	175	-2.29	0.103
	Grassland - Forest	-0.001	175	-0.32	0.988
	Settlement - Forest	-0.001	175	-0.03	0.999
	Settlement - Grassland	0.001	175	0.26	0.994

Supplementary Table 3. The output of SIMBA analysis. The analysis is testing slope differences by calculating the statistical inference of the difference in slope between two regression lines, and adjusting for the multiple comparisons with Bonferroni-Hochberg method. Comparisons are made between body size of arthropods in each local land-use type. Communities with different Hill numbers are separated (q=0, q=1, q=2). Bold letters indicate a significant effect ($p < 0.05$).

Hill-nr.	Land-use	Comparison *body size*	Difference in slope	Adjusted p (BH)
q=0	Settlement	Large - Medium	1.8E-05	0.924
		Large - Small	6.4E-05	0.108
		Small - Medium	4.6E-05	0.243
	Arable land	Large - Medium	7.6E-06	0.999
		Large - Small	3.1E-05	0.645
		Small - Medium	2.4E-05	0.681
	Grassland	Large - Medium	7.9E-06	0.999
		Large - Small	3.3E-05	0.342
		Small - Medium	2.5E-05	0.516
	Forest	Large - Medium	1.1E-05	0.999
		Large - Small	3.8E-05	0.189
		Small - Medium	2.7E-05	0.363
q=1	Settlement	Large - Medium	4.1E-05	0.468
		Large - Small	8.0E-05	0.057
		Small - Medium	4.0E-05	0.408
	Arable land	Large - Medium	5.1E-05	0.324
		Large - Small	3.6E-05	0.558
		Small - Medium	1.5E-05	0.999
	Grassland	Large - Medium	1.1E-05	0.999
		Large - Small	2.5E-05	0.594
		Small - Medium	3.6E-05	0.312
	Forest	Large - Medium	9.9E-06	0.999
		Large - Small	4.0E-05	0.264
		Small - Medium	5.0E-05	0.066
q=2	Settlement	Large - Medium	7.3E-05	0.102
		Large - Small	8.8E-05	0.036
		Small - Medium	1.6E-05	0.999
	Arable land	Large - Medium	1.0E-04	0.009
		Large - Small	3.1E-05	0.726
		Small - Medium	7.0E-05	0.045
	Grassland	Large - Medium	6.0E-05	0.063
		Large - Small	1.0E-06	0.999
		Small - Medium	5.9E-05	0.096
	Forest	Large - Medium	4.3E-05	0.210
		Large - Small	4.6E-05	0.132
		Small - Medium	8.8E-05	0.003

Supplementary Table 4. The output of SIMBA analysis. The analysis is testing slope differences by calculating the statistical inference of the difference in slope between two regression lines, and adjusting for the multiple comparisons with Bonferroni-Hochberg method. Comparisons are made between mobility of arthropods in each local land-use type. Communities with different Hill numbers are separated (q=0, q=1, q=2). Bold letters indicate a significant effect ($p < 0.05$).

Hill-nr.	Land-use	Comparison *mobility*	Difference in slope	Adjusted p (BH)
q=0	Settlement	High - Intermediate	7.4E-06	0.999
		Low - High	8.0E-05	0.162
		Low - Intermediate	7.3E-05	0.318
	Arable land	High - Intermediate	7.7E-05	0.054
		Low - High	1.1E-04	0.072
		Low - Intermediate	3.2E-05	0.906
	Grassland	High - Intermediate	5.7E-05	0.144
		Low - High	1.6E-04	0.003
		Low - Intermediate	2.1E-04	0.003
	Forest	High - Intermediate	6.0E-05	0.033
		Low - High	7.3E-05	0.024
		Low - Intermediate	1.3E-05	0.999
q=1	Settlement	High - Intermediate	2.6E-05	0.798
		Low - High	3.7E-05	0.732
		Low - Intermediate	1.1E-05	0.999
	Arable land	High - Intermediate	8.3E-05	0.039
		Low - High	1.0E-04	0.050
		Low - Intermediate	2.0E-05	0.999
	Grassland	High - Intermediate	5.2E-05	0.207
		Low - High	1.4E-04	0.003
		Low - Intermediate	1.9E-04	0.003
	Forest	High - Intermediate	7.0E-05	0.018
		Low - High	4.6E-05	0.219
		Low - Intermediate	2.4E-05	0.699
q=2	Settlement	High - Intermediate	3.8E-05	0.519
		Low - High	5.2E-06	0.999
		Low - Intermediate	3.3E-05	0.546
	Arable land	High - Intermediate	7.0E-05	0.093
		Low - High	3.7E-05	0.627
		Low - Intermediate	3.3E-05	0.633
	Grassland	High - Intermediate	4.0E-05	0.354
		Low - High	3.9E-05	0.339
		Low - Intermediate	7.9E-05	0.096
	Forest	High - Intermediate	7.9E-05	0.003
		Low - High	1.2E-05	0.999
		Low - Intermediate	6.7E-05	0.057

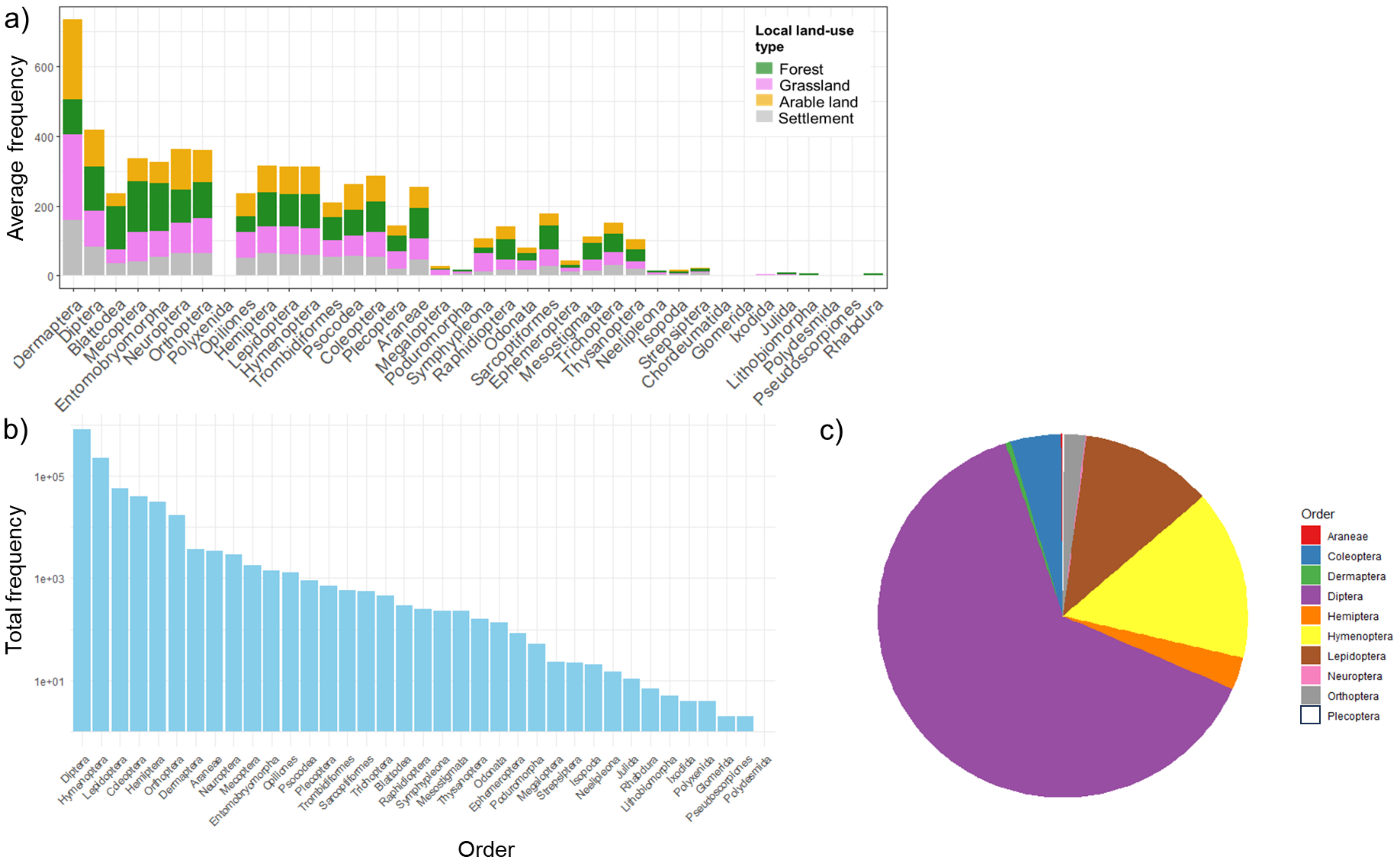

Supplementary Figure.1. Figure showing broad taxonomic summaries of the recovered arthropod species. The graphs show the (a) average frequencies of recovered BIN species across taxonomic orders per study plot, i.e. possible 0 – 16 multiplied by 35-55 plots of various land-use category (forest=green, grassland=purple, arable land = golden, settlement=grey); and (b) the total frequencies of recovered BIN species across taxonomic orders on all study plots of LandKlif. Y-axis is plotted on a log-scale. The pie chart (c) shows the total reads of BIN species across 10 taxonomic orders with the highest number of reads.

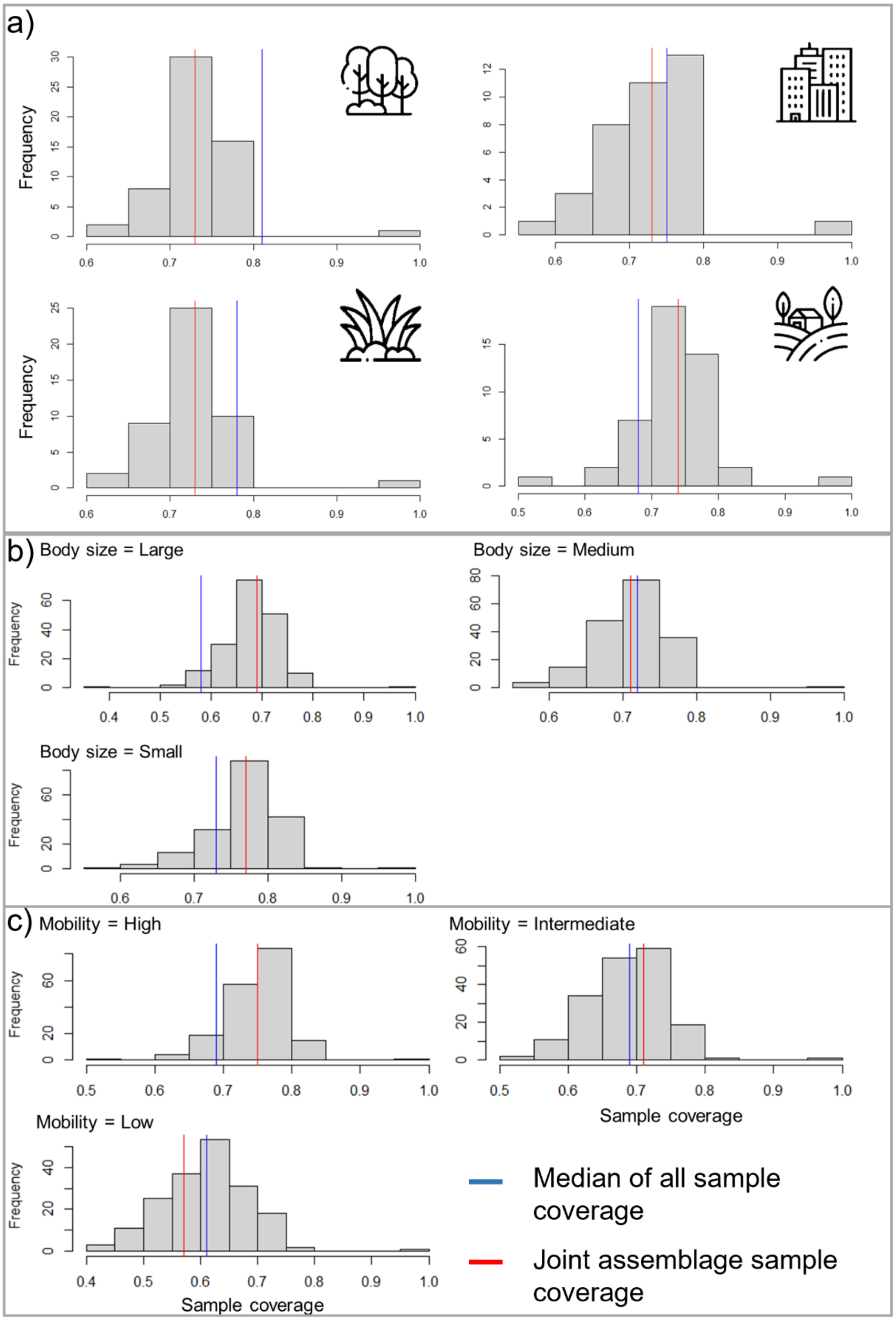

Supplementary Figure 2. Sample coverage of arthropod samples. (a) Sample coverage in different land-use types, sample coverage amongst species with different (b) body size categories and (c) species with different mobility categories. Used sample coverage to calculate community similarity for land-use types was 0.8, and for body size and mobility 0.7. Blue lines indicate overall median, red lines show the joint assemblage sample coverage.

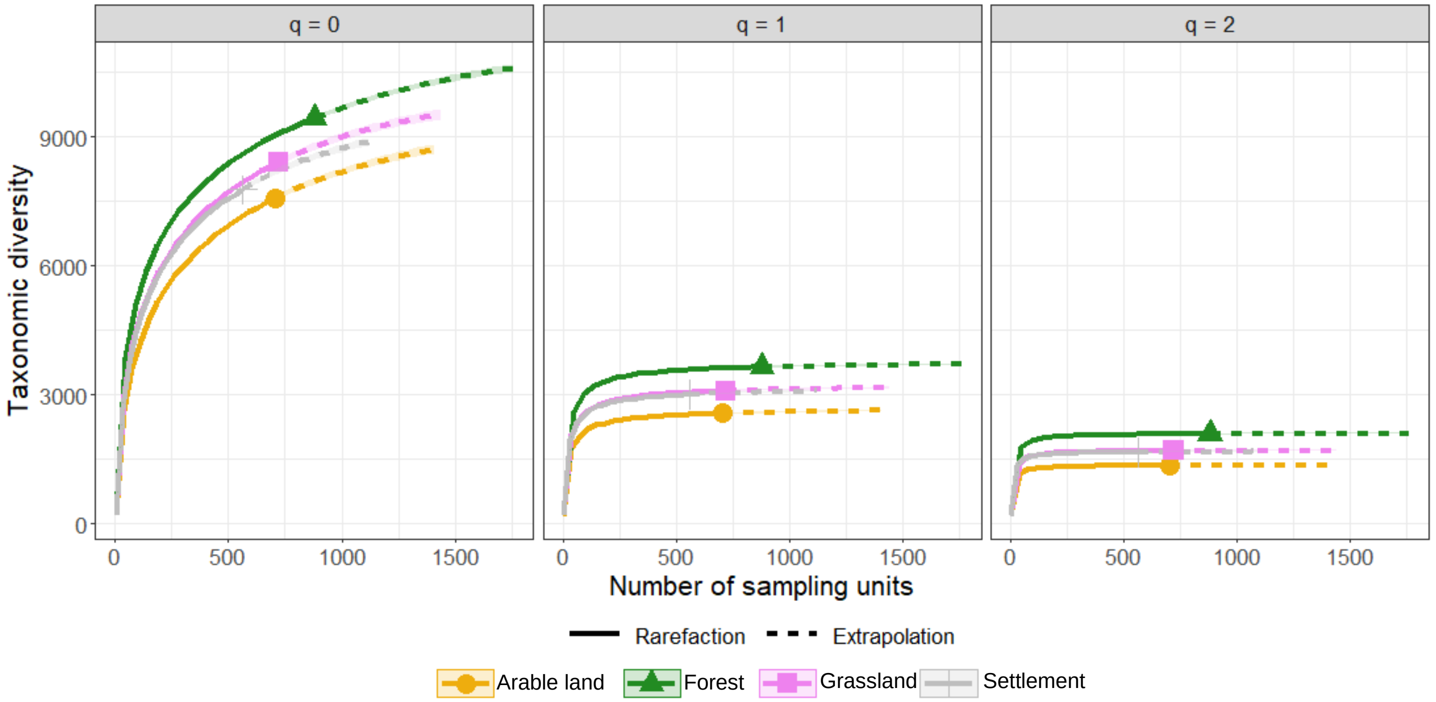

Supplementary Figure 3. Graph showing number of observed species at each land use type (golden=arable land, green=forest, purple=managed grassland, grey=settlement). Calculations were based on 179 study plots and ~12k arthropod species. Solid lines indicate rarefication, and dashed lines indicate extrapolation in estimating the observed species value based on the number of sampling units.

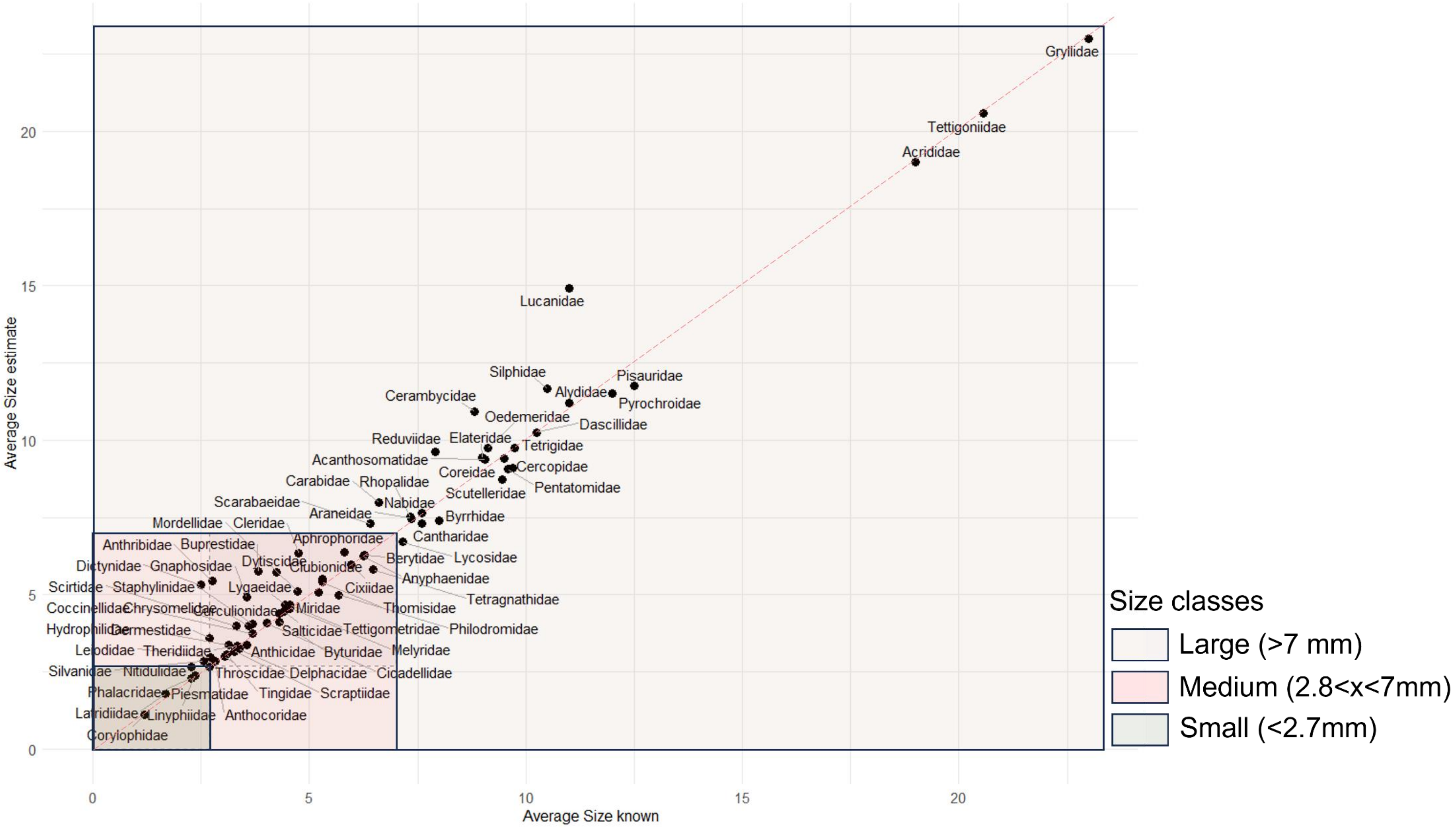

Supplementary Figure 4. The figure shows the relationship of body size estimates and true average body sizes of 71 arthropod families. The relationship was established based on species-specific data from Gossner et al., 2015. “A summary of eight traits of Coleoptera, Hemiptera, Orthoptera and Araneae, occurring in grasslands in Germany”, Sci Data 2, 150013. https://doi.org/10.1038/sdata.2015.13. Boxes indicate the size classes (green = small, red = medium, brick = large) used in the current paper based on estimates, and the red dashed line indicates the goodness of match between the two datasets (estimated and real sizes).
