# Supplementary material for: Distance-decay reveals contrasting effects of land-use types on arthropod community homogenisation
Source: Nat Commun. 2026 Jan 15;17:763. doi: 10.1038/s41467-025-67612-9 (PMC12820165; doi:10.1038/s41467-025-67612-9)
Supplement: Supplementary file 2 — Description of Additional Supplementary Information [file 41467_2025_67612_MOESM2_ESM.pdf]

Description of Additional Supplementary Information

Supplementary Data 1:

Table showing the used body size and mobility categories. Sources of the estimations can be found in the "source" column. Column E shows with an "+" if estimations were made using only the species or genus present in the dataset.
