# Supplementary material for: Distance-decay reveals contrasting effects of land-use types on arthropod community homogenisation
Source: Nat Commun. 2026 Jan 15;17:763. doi: 10.1038/s41467-025-67612-9 (PMC12820165; doi:10.1038/s41467-025-67612-9)
Supplement: Supplementary file 4 — Reporting Summary [file 41467_2025_67612_MOESM4_ESM.pdf]

nature portfolio

Corresponding author(s): Orsi Decker

Last updated by author(s): Sep 12, 2025

Reporting Summary

Nature Portfolio wishes to improve the reproducibility of the work that we publish. This form provides structure for consistency and transparency in reporting. For further information on Nature Portfolio policies, see our Editorial Policies and the Editorial Policy Checklist.

Statistics

For all statistical analyses, confirm that the following items are present in the figure legend, table legend, main text, or Methods section.

n/a	Confirmed	
☐	☒	The exact sample size (*n*) for each experimental group/condition, given as a discrete number and unit of measurement
☐	☒	A statement on whether measurements were taken from distinct samples or whether the same sample was measured repeatedly
☐	☒	The statistical test(s) used AND whether they are one- or two-sided<br>*Only common tests should be described solely by name; describe more complex techniques in the Methods section.*
☐	☒	A description of all covariates tested
☐	☒	A description of any assumptions or corrections, such as tests of normality and adjustment for multiple comparisons
☐	☒	A full description of the statistical parameters including central tendency (e.g. means) or other basic estimates (e.g. regression coefficient) AND variation (e.g. standard deviation) or associated estimates of uncertainty (e.g. confidence intervals)
☐	☒	For null hypothesis testing, the test statistic (e.g. *F*, *t*, *r*) with confidence intervals, effect sizes, degrees of freedom and *P* value noted<br>*Give P values as exact values whenever suitable.*
☒	☐	For Bayesian analysis, information on the choice of priors and Markov chain Monte Carlo settings
☒	☐	For hierarchical and complex designs, identification of the appropriate level for tests and full reporting of outcomes
☒	☐	Estimates of effect sizes (e.g. Cohen's *d*, Pearson's *r*), indicating how they were calculated

Our web collection on statistics for biologists contains articles on many of the points above.

Software and code

Policy information about availability of computer code

Data collection	No software was used to collect the data.
Data analysis	Data analysis was done using The R Foundation for Statistical Computing, version 4.3.1. Packages used: iNEXT.beta3D, FD, Simba, lme4, multcomp, ggplot2 (+ dependencies: devtools, iNEXT.3D, snowfall, vegan, fossil) .<br>Code is available at figshare together with the used data: https://doi.org/10.6084/m9.figshare.25941463.v3

For manuscripts utilizing custom algorithms or software that are central to the research but not yet described in published literature, software must be made available to editors and reviewers. We strongly encourage code deposition in a community repository (e.g. GitHub). See the Nature Portfolio guidelines for submitting code & software for further information.

Data

Policy information about availability of data

All manuscripts must include a data availability statement. This statement should provide the following information, where applicable:

- Accession codes, unique identifiers, or web links for publicly available datasets
- A description of any restrictions on data availability
- For clinical datasets or third party data, please ensure that the statement adheres to our policy

Data and used analysis are available at https://doi.org/10.6084/m9.figshare.25941463.v3

Research involving human participants, their data, or biological material

Policy information about studies with human participants or human data. See also policy information about sex, gender (identity/presentation), and sexual orientation and race, ethnicity and racism.

Reporting on sex and gender	*Use the terms sex (biological attribute) and gender (shaped by social and cultural circumstances) carefully in order to avoid confusing both terms. Indicate if findings apply to only one sex or gender; describe whether sex and gender were considered in study design; whether sex and/or gender was determined based on self-reporting or assigned and methods used. Provide in the source data disaggregated sex and gender data, where this information has been collected, and if consent has been obtained for sharing of individual-level data; provide overall numbers in this Reporting Summary. Please state if this information has not been collected. Report sex- and gender-based analyses where performed, justify reasons for lack of sex- and gender-based analysis.*
Reporting on race, ethnicity, or other socially relevant groupings	*Please specify the socially constructed or socially relevant categorization variable(s) used in your manuscript and explain why they were used. Please note that such variables should not be used as proxies for other socially constructed/relevant variables (for example, race or ethnicity should not be used as a proxy for socioeconomic status). Provide clear definitions of the relevant terms used, how they were provided (by the participants/respondents, the researchers, or third parties), and the method(s) used to classify people into the different categories (e.g. self-report, census or administrative data, social media data, etc.) Please provide details about how you controlled for confounding variables in your analyses.*
Population characteristics	*Describe the covariate-relevant population characteristics of the human research participants (e.g. age, genotypic information, past and current diagnosis and treatment categories). If you filled out the behavioural & social sciences study design questions and have nothing to add here, write "See above."*
Recruitment	*Describe how participants were recruited. Outline any potential self-selection bias or other biases that may be present and how these are likely to impact results.*
Ethics oversight	*Identify the organization(s) that approved the study protocol.*

Note that full information on the approval of the study protocol must also be provided in the manuscript.

Field-specific reporting

Please select the one below that is the best fit for your research. If you are not sure, read the appropriate sections before making your selection.

☐ Life sciences ☐ Behavioural & social sciences ☒ Ecological, evolutionary & environmental sciences

For a reference copy of the document with all sections, see nature.com/documents/nr-reporting-summary-flat.pdf

Ecological, evolutionary & environmental sciences study design

All studies must disclose on these points even when the disclosure is negative.

Study description	We studied arthropod communities collected by Malaisetraps and identified by Metabarcoding in 4 different land-use types across Germany.
Research sample	Our database included 1432 arthropod samples (179 study plots with 8 time periods), which gained 11387 individual arthropod taxonomic groups (BINs, thereafter 'species') via metabarcoding analysis
Sampling strategy	We collected data based on a stratified selected set of different habitats and climate conditions based on GIS analyses reported in Redlich et al (doi.org/10.1101/2021.03.05.434036)
Data collection	Malaise traps were emptied every 14 days from May to August 2019. For metabarcoding we first splitted in larger and smaller individuals and sequenced separately to increase the detectability of small species.
Timing and spatial scale	Sampling was done from May to August 2019 on 179 plots, covering a ~ 1000 m elevational gradient with a spatial extension of about 400 km.
Data exclusions	We excluded samples from traps which have been damaged or crashed.
Reproducibility	Our study is based on public data and open R code to allow full reproducibility of all results and graphs.
Randomization	Not applicapble
Blinding	Blinding is not possible in setting up insect traps (doi.org/10.1101/2021.03.05.434036)

Did the study involve field work? ☒ Yes ☐ No

Field work, collection and transport

Field conditions	We set up the Malaise Traps in a collaborative design (see Redlich et al doi.org/10.1101/2021.03.05.434036).
Location	The sampling area is in Central Europe, in the federal state Bavaria in South Eastern Germany.
Access & import/export	The material was collected with the permission of the nature conservation authorities of the governments Upper Franconia, Lower Franconia, Middle Franconia, Lower Bavaria, Upper Bavaria, Swabia, Upper Palatinate.
Disturbance	No disturbances were reported

Reporting for specific materials, systems and methods

We require information from authors about some types of materials, experimental systems and methods used in many studies. Here, indicate whether each material, system or method listed is relevant to your study. If you are not sure if a list item applies to your research, read the appropriate section before selecting a response.

Materials & experimental systems

n/a	Involved in the study	
☒	☐	Antibodies
☒	☐	Eukaryotic cell lines
☒	☐	Palaeontology and archaeology
☐	☒	Animals and other organisms
☒	☐	Clinical data
☒	☐	Dual use research of concern
☒	☐	Plants

Methods

n/a	Involved in the study	
☒	☐	ChIP-seq
☒	☐	Flow cytometry
☒	☐	MRI-based neuroimaging

Animals and other research organisms

Policy information about studies involving animals; ARRIVE guidelines recommended for reporting animal research, and Sex and Gender in Research

Laboratory animals	NA
Wild animals	NA
Reporting on sex	NA
Field-collected samples	Malaise traps were used to capture invertebrates with the following dimensions: height front: 0.90 m; height rear: 0.60 m; length: 1.60 m, with 80% ethanol as preserving solution.
Ethics oversight	NA

Note that full information on the approval of the study protocol must also be provided in the manuscript.

Plants

Seed stocks	NA
Novel plant genotypes	NA
Authentication	NA
